# 单中心85例初治滤泡淋巴瘤临床特征、预后分析及老年综合评估

**DOI:** 10.3760/cma.j.cn121090-20230815-00067

**Published:** 2024-03

**Authors:** 晶晶 尹, 龙 钱, 洁菲 白, 茹 冯, 江涛 李, 婷 王, 春丽 张, 辉 刘

**Affiliations:** 北京医院血液科、国家老年医学中心、中国医学科学院老年医学研究院，北京 100730 Department of Hematology, Beijing Hospital, National Center of Gerontology, Institute of Geriatric Medicine, Chinese Academy of Medical Science, Beijing 100730, China

**Keywords:** 淋巴瘤, 滤泡性, 生存分析, 老年综合评估, Lymphoma, Follicular, Survival analysis, Comprehensive geriatric assessment

## Abstract

**目的:**

探讨初治滤泡淋巴瘤（FL）患者的临床特征与预后，以及老年综合评估（comprehensive geriatric assessment，CGA）对国内年龄≥60岁FL患者的预后价值。

**方法:**

收集2011年8月至2022年6月收治的85例初诊FL患者临床资料及预后情况。统计分析患者临床特征、实验室指标、治疗疗效、生存及预后因素，使用多种老年评估工具对患者预后分层。

**结果:**

①FL患者多为中老年起病，中位发病年龄59（20～87）岁，其中年龄≥60岁者41例（48.2％），男女比例为1∶1.36。77.6％的患者诊断时Ann Arbor分期为Ⅲ～Ⅳ期，伴有B症状17例（20.0％），骨髓侵犯最常见（34.1％）。②71例患者接受一线系统化疗和（或）免疫治疗，可评估疗效的68例患者中总缓解率为86.8％，完全缓解率为47.1％。其中17例患者（23.9％）在治疗2年内病情进展或复发，共计10例（14.1％）患者死亡。③R-CHOP治疗组生存分析示：中位随访52.9（10.2～138.8）个月，3年无进展生存（PFS）率及总生存（OS）率分别为85.2％和95.9％，5年PFS率及OS率为72.8％和88.8％。单因素分析年龄≥60岁（*HR*＝3.430，95％ *CI* 1.256～9.371，*P*＝0.016）、B症状（*HR*＝5.030，95％ *CI* 1.903～13.294，*P*＝0.001）、预后营养指数（PNI）<45.25（*HR*＝3.478，95％ *CI* 1.299～9.310，*P*＝0.013）、FL国际预后指数（FLIPI）高危（*HR*＝2.918，95％ *CI* 1.074～7.928，*P*＝0.036）、PRIMA预后指数（PRIMA-PI）高危（*HR*＝2.745，95％ *CI* 1.057～7.129，*P*＝0.038）是PFS的不良预后因素。POD24（*HR*＝9.160，95％ *CI* 1.202～69.830，*P*＝0.033）是OS的不良预后因素。多因素分析中年龄≥60岁（*HR*＝3.002，95％ *CI* 1.014～8.889，*P*＝0.047），B症状（*HR*＝3.810，95％ *CI* 1.052～13.801，*P*＝0.042）能独立预测PFS，未发现影响OS的独立预测因素。

**结论:**

FL多为中老年女性患者。年龄、B症状、PNI、FLIPI、PRIMA-PI、POD24是影响FL患者PFS和OS的重要因素。CGA对预测老年FL预后及指导治疗均可能具有一定价值。

## Abstract (en)

**Objective:**

To retrospectively analyze the clinical characteristics and prognosis of 85 newly diagnosed patients with follicular lymphoma（FL）, as well as the prognostic value of comprehensive geriatric assessment（CGA）in patients with FL aged ≥ 60 years old.

**Methods:**

The clinical data and prognosis of 85 newly diagnosed FL patients admitted from August 2011 to June 2022 were collected. The clinical features, laboratory indicators, therapeutic efficacy, survival and prognostic factors of patients were statistically analyzed, and the prognosis of patients was stratified using various geriatric assessment tools.

**Results:**

① The patients with FL were mostly middle-aged and older, with a median age of 59（20−87）years, including 41 patients（48.2%）aged ≥60 years. The ratio of male to female was 1∶1.36. Overall, 77.6% of the patients were diagnosed with Ann Arbor stage Ⅲ−Ⅳ, and 17 cases（20.0%）were accompanied by B symptoms. Bone marrow involvement was the most common（34.1%）. ②Overall, 71 patients received immunochemotherapy. The overall response rate was 86.6%, and the complete recovery rate was 47.1% of 68 evaluated patients. Disease progression or relapse in the first 2 years was observed in 23.9% of the patient. Overall, 14.1% of the patients died during follow-up. ③Of the 56 patients receiving R-CHOP-like therapies, the 3-year and 5-year progression-free survival（PFS）rates were 85.2% and 72.8%, respectively, and the 3-year and 5-year overall survival（OS）rates were 95.9% and 88.8%, respectively. The univariate analysis showed that age ≥60 years old（*HR*＝3.430, 95% *CI* 1.256–9.371, *P*＝0.016）, B symptoms（*HR*＝5.030, 95% *CI* 1.903–13.294, *P*＝0.016）, Prognostic Nutritional Index（PNI）<45.25（*HR*＝3.478, 95% *CI* 1.299–9.310, *P*＝0.013）, Follicular Lymphoma International Prognostic Index（FLIPI）high-risk（*HR*＝2.918, 95% *CI* 1.074–7.928, *P*＝0.036）, and PRIMA-prognostic index（PRIMA-PI）high-risk（*HR*＝2.745, 95% *CI* 1.057–7.129, *P*＝0.038）significantly predicted PFS. Moreover, age ≥60 years old and B symptoms were independent risk factors for PFS. Progression of disease within 24 months（POD24）significantly predicted OS in the univariate analysis.

**Conclusion:**

FL is more common among middle-aged and older women. Age, B symptoms, PNI score, FLIPI high-risk, PRIMA-PI high-risk, and POD24 influenced PFS and OS. The CGA can be used for treatment selection and risk prognostication in older patients with FL.

滤泡淋巴瘤（FL）是一类生发中心B细胞起源的淋巴造血组织肿瘤性疾病，是常见的惰性非霍奇金淋巴瘤（NHL）之一，在西方国家占NHL 22％～35％，疾病异质性高，患者中位年龄为60～65岁[1]–[3]。国内病例报道FL发病率稍低，占所有NHL患者8.1％～23.5％，中位年龄为49～53岁[4]–[7]。除FL 3B级按照侵袭性淋巴瘤治疗外，FL作为惰性淋巴瘤自然病程较长，总体预后较好，但易复发需多线治疗，绝大多数不可治愈，FL早期复发，严重损害其总生存（OS）。目前国际上已提出针对FL预后的多种模型，如FL国际预后指数（follicular lymphoma international prognostic index，FLIPI）、FLIPI-2、PRIMA预后指数（PRIMA-prognostic index，PRIMA-PI）评分系统等[8]–[11]。年龄是FL的重要预后因素，随着人口老年化趋势，老年FL治疗抉择也成为一大难题。老年综合评估（CGA）是评估老年肿瘤患者的重要手段，近年来逐渐广泛用于指导弥漫大B细胞淋巴瘤（DLBCL）治疗方案的抉择及预后。但是CGA用于FL国外报道较少，国内尚无报道。本研究通过回顾性分析北京医院初治FL患者，旨在分析其临床特征及预后关系。同时进一步阐明CGA在中国FL患者中的可行性及有效性，为今后进一步研究提供基础。

## 病例与方法

1. 研究人群：纳入2011年8月至2022年6月于北京医院确诊的成人FL患者，排除FL 3B级及伴大B细胞转化患者，其中具有较为完整基线资料的患者共85例。所有患者临床及实验室检查符合WHO（2016）造血与淋巴组织肿瘤分类制定的诊断标准。根据中心母细胞数量确定FL分级：1～2级、3A级。本研究为回顾性研究，通过北京医院医学伦理委员会审批（批件号：2022BJYYEC-238-02），免除签署知情同意书。

2. 病例资料：根据患者影像学及骨髓检查结果确定Ann Arbor分期，记录患者基线资料如年龄、性别、美国东部肿瘤协作组（ECOG）评分、LDH、β_2_微球蛋白水平，根据既往文献计算患者FLIPI及FLIPI-2、PRIMA-PI、预后营养指数（PNI）[8]–[10],[12]。PNI＝白蛋白（g/L）+5×淋巴细胞计数（×10^9^/L）。回顾性分析患者人口学特征、临床特征、一线治疗方案、治疗疗效及远期生存等。CGA涵盖内容包括躯体功能状况、合并症、心理状态、认知功能、营养状态、社会经济状态等。本中心对于年龄≥60岁患者评估其日常生活活动量表（activities of daily living scale，ADL）、工具性日常生活活动量表（instrumental activities of daily living scale，IADL）及老年疾病累计评分法（cumulative illness rating scale-geriatrics，CIRS-G）。同时基于既往文献，根据不同评分体系如简化CGA评分体系、年龄-合并症-白蛋白（age, comorbidities and albumin index，ACA）指数、IACA（IADL，ACA）指数将患者分组[13]–[15]，分析不同组患者疗效及预后情况。

3. 治疗方案：本组患者中4例治疗方案不详，有较完整随访资料共计81例。8例患者因无症状低肿瘤负荷采用观察等待治疗，1例患者采用局部微波消融治疗，1例高龄患者拒绝化疗仅使用激素治疗。其余71例患者一线治疗接受系统治疗：其中60例患者接受含R-CHOP（利妥昔单抗+环磷酰胺+多柔比星+长春新碱+泼尼松）方案，5例接受BR（利妥昔单抗+苯达莫司汀）方案，1例采用FCR（利妥昔单抗+氟达拉滨+环磷酰胺）方案，5例患者采用无化疗方案：R（利妥昔单抗）单药或R2（利妥昔单抗+来那度胺）方案；维持治疗采用靶向CD20单抗（利妥昔单抗或奥妥珠单抗）治疗。

4. 随访与评估：所有患者随访至2023年8月，随访资料来源于病历资料、门诊或电话记录。OS期为疾病确诊时间至死亡时间或末次随访时间。无进展生存（PFS）期为疾病确诊时间至出现病情复发、进展或任何原因死亡的时间或末次随访。POD24为从疾病确诊至24个月内出现疾病进展或复发。总缓解率（ORR）包括完全缓解（CR）率、部分缓解（PR）率。疗效标准参照既往Lugano标准[16]定义诊断疾病缓解、进展或复发。

5. 统计学处理：计数资料使用例（％）描述。计量资料使用*M*（范围）描述。统计采用SPSS 26.0软件及决策链统计软件进行分析。对于缺失值通过多重插补连续5次，选取最后一次插补结果填充。使用受试者工作特征曲线确定PNI指数的截点值。多组之间分类变量数据比较采用卡方检验或Fisher精确检验。单因素分析采用Kaplan-Meier曲线的Log-rank法，多因素分析采用Cox比例风险回归模型，*P*<0.05为差异有统计学意义。

## 结果

1. 基线特征：如表1所示，85例FL患者中位年龄59（29 ~ 87）岁，其中年龄≥60岁者41例（48.2％）。男36例，女49例。病理分型中3A级30例（35.3％）。60例（70.6％）以淋巴结肿大为首发症状。11例（12.9％）起病时ECOG评分≥2分，Ann Arbor Ⅲ～Ⅳ期有66例（77.6％），伴有B症状17例（20.0％）。31例（36.6％）HGB<120 g/L，7例（8.2％）LDH升高，62例（77.5％）β_2_微球蛋白水平升高。29例（34.1％）存在骨髓受累。58例（68.2％）淋巴结受累数目≥5个，10例（11.8％）淋巴结最大直径>6 cm。除骨髓外，结外受累部位中最常见包括脾脏27例（31.8％），骨10例（11.8％），肝脏、胃肠道各8例（9.4％），其他部位有腮腺、肺、甲状腺等。31例（36.5％）结外受累部位≥2个。患者依据FLIPI、FLIPI-2、PRIMA-PI危险度分层见表1。

**表1:** 85例滤泡淋巴瘤患者的基线特征

临床特征	例数（%）
中位年龄[岁，*M*（范围）]	59（29～87）
年龄≥60岁	41（48.2）
男性	36（42.4）
组织学分级	
1～2级	55（64.7）
3A级	30（35.3）
首发症状：淋巴结肿大	60（70.6）
ECOG评分≥2分	11（12.9）
Ann Arbor Ⅲ～Ⅳ期	66（77.6）
B症状	17（20.0）
HGB<120 g/L	31（36.5）
LDH升高	7（8.2）
β_2_微球蛋白水平升高^a^	62（77.5）
骨髓受累	29（34.1）
淋巴结受累区域≥5个	58（68.2）
淋巴结最大直径>6 cm	10（11.8）
结外器官受累≥2个	31（36.5）
FLIPI评分	
低危	16（18.8）
中危	34（40.0）
高危	35（41.1）
FLIPI-2评分^b^	
低危	23（28.4）
中危	30（37.0）
高危	28（34.6）
PRIMA-PI评分^a^	
低危	42（52.5）
中危	15（18.8）
高危	23（28.7）

注 ^a^ 80例可供分析；^b^ 81例患者可供分析。ECOG：美国东部肿瘤协作组；FLIPI：滤泡淋巴瘤国际预后指数；FLIPI-2：滤泡淋巴瘤国际预后指数2；PRIMA-PI：PRIMA预后指数

2. 治疗方案及疗效：85例患者中，接受一线全身系统治疗共71例。100％患者接受CD20靶向治疗，最常用治疗方案为R-CHOP方案（60例），其他治疗方案如BR/FCR（6例）、R/R2（5例）等。71例患者中，64例（90.1％）患者接受4个周期以上治疗，55例（77.5％）患者接受6个周期以上治疗。免疫化疗后56例（78.8％）患者接受靶向CD20维持治疗。68例患者可评估一线治疗疗效，ORR为86.8％（59/68），初治CR率为47.1％（32/68）。因为大部分患者接受R-CHOP样方案化疗，因此暂不比较不同方案间疗效。

3. 疾病转归：85例患者随访期间共29例出现病情进展或复发。接受一线化疗和（或）免疫治疗的71例患者进展或复发共24例，17例（23.9％）患者在治疗2年内病情进展或复发。2例（2.8％）患者化疗后转化为DLBCL。观察等待或局部治疗组进展复发5例。71例患者中共10例（14.1％）死亡，2例化疗后病情相对稳定，但因合并危重型新冠肺炎出现死亡。1例患者淋巴瘤合并肠道肿瘤及肛周脓肿，化疗耐受性差，后因原发病及感染加重出现死亡，7例患者因原发病加重出现死亡。观察等待和局部治疗组2例出现原发病加重死亡。

4. 生存分析：选取病例数最多的R-CHOP方案治疗组（60例），其中4例患者一线化疗治疗仅接受1～2个周期。因此将接受≥4个周期的56例患者纳入分析，其中52例患者接受6～8个周期一线免疫化疗方案。一线免疫化疗治疗后49例（87.5％）患者达到部分或完全缓解，45例患者接受靶向CD20维持治疗，中位疗程7（2～8）个周期，中位维持时间为21.3（6.6～25.6）个月。使用受试者工作特征曲线确定PNI截点值分别为45.25。中位随访52.9（10.2～138.8）个月，3年PFS率及OS率为85.2％和95.9％，5年PFS率及OS率为72.8％和88.8％。考虑到病例数较少，FLIPI评分、FLIPI-2和PRIMA-PI评分均分为低中危组和高危组。生存分析如下：单因素分析结果显示，年龄≥60岁、B症状、PNI<45.25、FLIPI高危组、PRIMA-PI高危是PFS不良预后因素（*P*<0.05）。POD24是患者OS的不良预后因素（*P*<0.05）。多因素分析结果显示，年龄≥60岁（*HR* 3.002，95％*CI* 1.014～8.889，*P*＝0.047），B症状（*HR* 3.810，95％*CI* 1.052～13.801，*P*＝0.042）能独立预测PFS，未发现影响OS的独立预测因素（表2）。

**表2:** 56例接受≥4个周期R-CHOP方案治疗的滤泡淋巴瘤患者的单因素及多因素生存分析

变量	单因素分析				多因素分析			
	PFS		OS		PFS		OS	
	*HR*（95%*CI*）	*P*值	*HR*（95%*CI*）	*P*值	*HR*（95%*CI*）	*P*值	*HR*（95%*CI*）	*P*值
年龄≥60岁	3.430(1.256~9.371)	0.016	8.557(0.932~78.536)	0.058	3.002(1.014~8.889)	0.047	7.362(0.598~90.584)	0.119
性别：男	0.455(0.165~1.256)	0.128	0.312(0.051~1.916)	0.209				
病理分级：3级	1.100(0.404~2.996)	0.852	3.370(0.556~20.411)	0.186				
B症状	5.030(1.903~13.294)	0.001	6.196(0.996~38.545)	0.051	3.810(1.052~13.801)	0.042	4.697(0.494~44.698)	0.178
HGB<120 g/L	1.712(0.660~4.440)	0.269	1.060(0.176~6.383)	0.949				
β_2_微球蛋白升高	1.565(0.449~5.456)	0.482	1.302(0.145~11.713)	0.814				
LDH升高	2.143(0.596~7.707)	0.243	1.852(0.206~16.635)	0.582				
骨髓受累	1.119(0.412~3.039)	0.825	1.557(0.259~9.374)	0.629				
结外器官受累≥2个	0.935(0.342~2.560)	0.896	2.718(0.448~16.491)	0.277				
分期：Ⅲ/Ⅳ	1.283(0.293~5.622)	0.741	2.279×10^7^(0~inf)	0.999				
PNI<45.25	3.478(1.299~9.310)	0.013	5.618(0.909~34.729)	0.063				
FLIPI：高危	2.918(1.074~7.928)	0.036	1.982(0.329~11.934)	0.455				
FLIPI-2：高危	2.553(0.982~6.636)	0.054	2.937(0.489~17.633)	0.239				
PRIMA-PI：高危	2.745(1.057~7.129)	0.038	3.100(0.514~18.696)	0.217				
CD20单抗维持	0.828(0.236~2.908)	0.768	0.678(0.075~6.122)	0.730				
POD24			9.160(1.202~69.830)	0.033				

注 R-CHOP：利妥昔单抗+环磷酰胺+多柔比星+长春新碱+泼尼松；PFS：无进展生存；OS：总生存；PNI：预后营养指数；FLIPI：滤泡淋巴瘤国际预后指数；FLIPI-2：滤泡淋巴瘤国际预后指数-2；PRIMA-PI：PRIMA-预后指数；POD24：治疗2年内复发

5. 不同年龄患者基线特征及治疗对比：如表3所示，基于年龄将患者分为三组：<60岁，60～<80岁，≥80岁。从基线临床特征看，三组在ECOG评分（*P*<0.001）、PNI≥45.25（*P*＝0.024）、FLIPI评分高危（*P*＝0.048）、FLIPI-2评分高危（*P*＝0.001）构成比差异具有统计学意义。年龄≥80岁组ECOG评分<2比例明显高于其他两组（*P*<0.001），PNI更低（*P*＝0.024），FLIPI-2评分高危比例更高（*P*＝0.005）。FLIPI高危构成比三组差异具有统计学意义，但年龄≥80岁及60～<80岁两组在FLIPI高危构成比上差异无统计学意义。从治疗方案上看，年龄<60岁及60～<80岁两组患者治疗方案以R-CHOP方案为主，年龄≥80岁人群无化疗方案比例高于另外两组，三组患者的3年PFS率及3年OS率差异具有统计学意义（*P*＝0.001和*P*＝0.008），年龄≥80岁患者组非疾病相关死亡率高于其他两组，差异无统计学意义（*P*＝0.584）。

**表3:** 不同年龄的滤泡淋巴瘤患者的基线特征数据及组间比较

基线特征	<60岁（44例）	60～80岁（31例）	≥80岁（10例）	*P*值
年龄[岁，*M*（范围）]	51（29~59）	65（60~79）	84（80~87）	
B症状（例，无/有）	36/8	25/6	7/3	0.696
ECOG评分<2分（例，是/否）	42/2	28/3	5/5	<0.001
HGB<120 g/L（例，否/是）	28/16	22/9	4/6	0.209
LDH（例，正常/升高）	42/2	27/4	9/1	0.381
PNI≥45.25（例，否/是）	34/10	21/10	3/7	0.024
FLIPI评分（例，低中危/高危）	31/13	13/18	5/5	
FLIPI-2评分^a^（例，低中危/高危/缺失）	35/8/1	16/13/2	2/7/1	
PRIMAPI评分^b^（例，低中危/高危/缺失）	33/9/2	19/10/2	5/4/1	
治疗方案（例数）				
R-COP/R-CHOP	36	19	5	
BR/FCR	2	3	1	
R/R2	0	1	4	
观察等待	2	6	0	
治疗不详	4	2	0	
3年PFS率（%）	89.5	66.2	46.7	0.001
3年OS率（%）	96.8	84.7	78.8	0.008
非疾病相关死亡率（%）	2.3	3.2	10.0	0.584

注 ^a^ 81例可供分析；^b^ 80例患者可供分析。ECOG：美国东部肿瘤协作组评分；PNI：预后营养因子；FLIPI：滤泡淋巴瘤国际预后因子；FLIPI-2：滤泡淋巴瘤国际预后因子-2；PRIMA-PI：PRIMA预后因子；R-CHOP：利妥昔单抗+环磷酰胺+多柔比星+长春新碱+泼尼松；BR：利妥昔单抗+苯达莫司汀；FCR：利妥昔单抗+氟达拉滨+环磷酰胺；R：利妥昔单抗；R2：利妥昔单抗+来那度胺；PFS：无进展生存；OS：总生存

6. 老年患者治疗及老年评估分析：本中心老年FL患者（即年龄≥60岁）共41例，基于不同老年评估标准如简化CGA评分体系、ACA指数和IACA指数将41例患者分组。依据简化CGA体系，适合组、不适合组、脆弱组分别有18、3和20例；依据ACA指数，预后好组、预后较好组、预后中等组、预后差组分别有21、12、6、2例；依据IACA指数，低危组、中危组、高危组分别有26、9、6例。41例患者中33例接受系统治疗，治疗方案分为足量治疗及减量治疗。足量治疗包括R-CHOP方案、BR化疗方案，减量治疗包括减量化疗（减量R-CHOP、BR化疗）、无化疗方案R/R2。简化CGA体系、ACA指数、IACA指数分类下，各组患者接受足量治疗、减量化疗及无化疗方案例数如表4所示。中位随诊时间为31.2（3.0～138.8）个月，20例（60.6％）患者出现3～4级血液学毒性，7例（21.2％）患者有3～4级感染事件，各有3例（9.0％）患者出现3～4级心功能不全、胃肠道反应等，1例患者化疗后有3～4级肝功能异常等。生存分析如图1所示，简化CGA评分下不适合/脆弱组患者PFS和OS期较适合组明显缩短（*P*＝0.001；*P*＝0.010）。亚组分析中，适合组患者中足量治疗PFS和OS有趋势优于减量治疗者，但差异无统计学意义；不适合/脆弱组中足量治疗和减量治疗患者PFS和OS差异无统计学意义，减量治疗者较长随访下OS有趋势优于足量治疗。如图2及图3所示，ACA指数分层下预后好/较好组与预后中等/差组PFS差异有统计学意义（*P*<0.001），但OS差异无统计学意义（*P*＝0.130）。IACA指数分层下低危和中高危两组PFS及OS差异无统计学意义（*P*＝0.148，*P*＝0.272）。

**表4:** 33例老年滤泡淋巴瘤患者一线治疗方案（例数）

评分体系	组别	例数	足量治疗	减量化疗	无化疗治疗
简化CGA体系	适合	12	10	2^a^	0
	不适合/脆弱	21	9	9	3
ACA指数	预后好/较好	25	18	5	2
	预后中等/差	8	1	6	1
IACA指数	低危	19	17	1	1
	中/高危	14	2	10	2

注 简化CGA：简化老年综合评估体系；ACA指数：年龄-合并症-白蛋白指数；IACA指数：基于工具性日常生活活动量表IADL，ACA指数计算；^a^适合组中，2例患者因个人原因自行要求减量化疗疗程

**图1:**
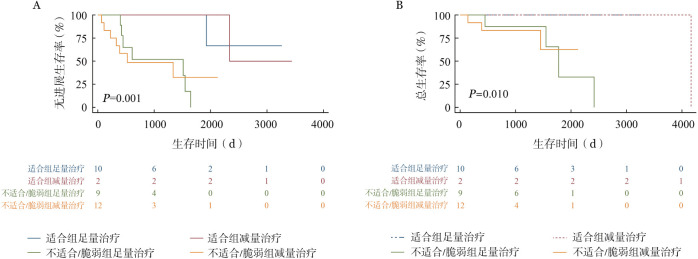
简化老年综合评估体系评分下适合组、不适合/脆弱组接受足量治疗、减量治疗的无进展生存（A）和总生存（B）曲线

**图2:**
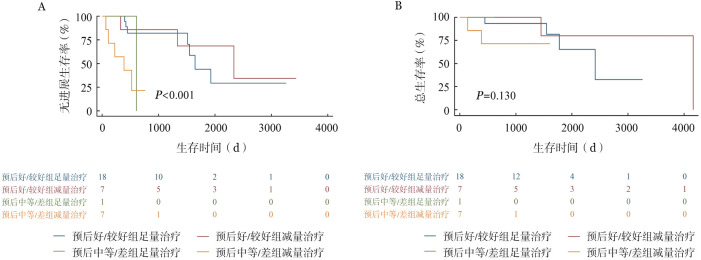
ACA指数评分下预后好/较好组、预后中等/差组接受足量治疗、减量治疗的无进展生存（A）和总生存（B）曲线 注 ACA指数：年龄-合并症-白蛋白指数

**图3:**
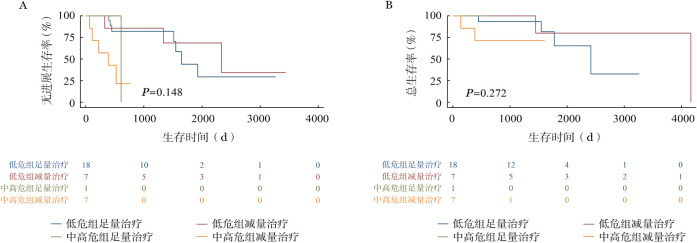
IACA指数评分下低危组、中高危组接受足量治疗、减量治疗的无进展生存（A）和总生存（B）曲线 注 IACA指数：基于工具性日常生活活动量表、年龄-合并症-白蛋白指数

## 讨论

FL作为常见惰性淋巴瘤之一，自然病程较长，易反复复发需多线治疗，绝大多数不可治愈。随着血液肿瘤诊断技术提高以及靶向药物利妥昔单抗的广泛应用，FL患者整体预后改善，中位生存时间可达15年以上，国外报道5年及10年OS率可达90％和80％[17]，而我国既往报道5年及10年OS 率分别为89％和75％[5],[18]–[19]。但高达20％ FL患者仍然出现2年内进展或复发，导致生存期缩短[10],[20]，其高异质性需要我们不断完善预后评估体系识别预后不良患者。本中心分析初诊FL患者预后危险因素，并探索CGA在老年FL患者中应用价值。

FL多见于老年群体且女性患者较多。在发病率及人口学特点方面，国内外略有不同。本中心患者中位年龄59岁，发病中位年龄低于西方国家（60～65岁），但高于国内既往报道（49～53岁）[1],[5]，可能由于本单位就诊患者年龄偏大。本中心FL患者11例（12.9％）起病时ECOG评分≥2，弱体能状态比例高于国内既往报道结果（1.8％～6％）[5],[18]。66例（77.6％）分期属于Ⅲ～Ⅳ期，62例（77.5％）患者存在β_2_微球蛋白升高（>1.8 mg/L）。据国内的一项研究纳入1 845例FL患者，骨髓受累比例低于西方国家（28％对29％～70％）[3]。但据另一项国内纳入288例FL患者的研究，骨髓受累比例占41.7％[21]。而本中心29例（34.1％）患者存在骨髓受累，未来需要扩大样本量研究。本研究人群其他基线特征如组织学分级、B症状比例、淋巴结受累数目、分期、生化指标如β_2_微球蛋白、LDH等与国内外既往结果大致相同[1],[5]。结外部位受累方面，我们发现最常见为骨髓，其次为脾脏，与国内既往报道一致[22]。

目前普遍认为年龄是FL患者预后不良因素之一。年龄作为重要因素被纳入广泛使用的FL预后评分指数FLIPI和FLIPI-2。Junlén等[23]发现年龄处于18～49岁FL患者10年OS率为92％，而年龄在50～59岁、60～69岁以及70岁以上患者的10年OS率分别为83％、78％和64％。然而，年龄对预后结局的影响究竟是因为高龄患者遗传学结果更差，或是因为治疗强度减低，还是因为严重并发症一直饱受争议。基于国外一项研究，年龄>70岁的NHL患者中61％存在严重并发症[24]。FL患者随着年龄增长基因突变数量增多，有报道称突变主要包括沉默突变以及对功能影响较小的错义突变[25]。研究质疑年龄预测患者预后的价值，并指出年龄>70岁FL患者生存期更短主要原因是随着年龄增长非疾病复发死亡病因增加，而不是疾病进展。研究提出在年龄因素外基于β_2_微球蛋白和骨髓是否受累建立新的模型PRIMA-PI。本中心发现年龄<60岁、60～80岁以及80岁以上患者中，年龄≥80岁患者ECOG 评分更差，PNI指数更低，FLIPI-2评分高危比例更高，FLIPI高危比例高于年龄<60岁患者；年龄≥ 80岁患者接受化疗方案比例更低，与Nabhan等[1]研究一致。本研究中年龄≥80岁组患者3年PFS率和3年OS率低于年龄<60岁及60～80岁患者（*P*<0.05），虽然三组中年龄≥80岁患者非疾病死亡比例更高，但差异无统计学意义，本中心验证了年龄是FL患者重要不良预后因素。

既往FL研究中已提出多种预后模型，除FLIPI、FLIPI-2、PRIMA-PI外，有基于分子遗传学基因突变m7-FLIPI等，此外还有基于外周血单核/淋巴细胞计数、PNI指数等模型，但仍需进一步验证[26]。PNI指数由宿主白蛋白与淋巴细胞计数计算得出，一定程度上反映宿主的营养状态。Ge等[12]研究发现PNI指数<44.3时FL患者治疗CR率显著下降，PNI指数与患者PFS、OS相关且独立预测患者OS。本中心发现年龄、B症状、PNI指数、FLIPI、PRIMA-PI评分与患者PFS相关，POD24是影响OS不良预后因素。多因素分析示年龄、B症状是影响PFS的独立危险因素。本研究未能发现FLIPI、FLIP2、PRIMA-PI对OS的预后价值，Ann Arbor分期Ⅲ/Ⅳ期相比于Ⅰ/Ⅱ期患者OS期短，可能与FL患者整体生存较好以及样本量较少有关。同时本中心为减少个别数据缺失数据量不足，采用统计学方法补齐数据，但仍可能带来误差。未来仍需扩大样本量，延长随访时间进一步研究。

CGA在急性白血病[27]、慢性淋巴细胞白血病[28]、多发性骨髓瘤[29]等血液肿瘤患者中都已证实可指导患者预后分层及治疗，但目前CGA无统一标准。近些年来意大利FIL研究组[13]、日本SOLT-J[14]先后提出简化CGA体系、ACA指数用于指导DLBCL分层治疗、可预测患者预后[30]。本中心在日本ACA指数基础上进行优化提出IACA指数，能够在老年DLBCL患者中预测患者疗效、PFS及OS，用于指导治疗决策[15]。意大利FIL研究组进一步提出简化老年评估sGA体系能独立预测DLBCL患者OS[31]。然而CGA在老年FL患者预后及辅助治疗决策等研究很少，且近几年鲜有报道。2012年一项回顾性研究分析303例年龄≥80岁NHL患者体能状态和合并症对预后的影响，其中包含34例FL患者。该研究多因素生存分析得出ADL受损是患者PFS和OS的独立预测因素[32]。本中心试图将CGA用于FL预后评估。通过应用简化CGA体系、ACA指数、IACA指数将接受治疗的33例老年患者FL分层，结果发现简化CGA评分中，一半以上患者评估属于不适合/脆弱组。适合组患者PFS及OS明显长于不适合/脆弱组，一定程度上反应简化CGA评分可用于预测预后。亚组分析中，不适合/脆弱组患者足量治疗未能带来PFS或OS获益，不良反应增多，OS有趋势短于减量治疗，表明依据CGA评分指导治疗方案抉择可能获益。本研究中ACA指数及IACA指数分层在预测患者生存价值劣于简化CGA评分，可能与病例数较少有关，未来需要在更大样本量人群中比较三种老年评估工具的预后预测价值。

综上所述，我国FL患者有独特的临床特征，总体预后较好。年龄、B症状、PNI指数、FLIPI、FLIPI-2、PRIMA-PI、POD24在预测患者生存存在一定价值。随着老年FL患者增多，CGA有望应用于临床指导老年FL患者预后及治疗选择，改善患者预后并尽可能减少治疗不良反应，未来需要设计临床试验及扩大样本量以进一步研究。
